# Optimization of a pretreatment and hydrolysis process for the efficient recovery of recycled sugars and unknown compounds from agricultural sweet sorghum bagasse stem pith solid waste

**DOI:** 10.7717/peerj.6186

**Published:** 2019-01-10

**Authors:** Ting-Ting Jiang, Yan Liang, Xiang Zhou, Zi-Wei Shi, Zhi-Jun Xin

**Affiliations:** 1 Institute of Modern Physics, Chinese Academy of Sciences, Lanzhou, Gansu, P.R. China; 2 University of Chinese Academy, Beijing, P.R. China; 3 School of Pharmacy, Lanzhou University, Lanzhou, Gansu, P.R. China; 4 Gansu Agricultural University, Lanzhou, Gansu, P.R. China

**Keywords:** Pith, Particle size, Integrated process, Enzymatic hydrolysis, Monosaccharide, Unknown compound

## Abstract

**Background:**

Sweet sorghum bagasse (SSB), comprising both a dermal layer and pith, is a solid waste generated by agricultural activities. Open burning was previously used to treat agricultural solid waste but is harmful to the environment and human health. Recent reports showed that certain techniques can convert this agricultural waste into valuable products. While SSB has been considered an attractive raw material for sugar extraction and the production of value-added products, the pith root in the SSB can be difficult to process. Therefore, it is necessary to pretreat bagasse before conventional hydrolysis.

**Methods:**

A thorough analysis and comparison of various pretreatment methods were conducted based on physicochemical and microscopic approaches. The responses of agricultural SSB stem pith with different particle sizes to pretreatment temperature, acid and alkali concentration and enzyme dosage were investigated to determine the optimal pretreatment. The integrated methods are beneficial to the utilization of carbohydrate-based and unknown compounds in agricultural solid waste.

**Results:**

Acid (1.5−4.5%, v/v) and alkali (5−8%, w/v) reagents were used to collect cellulose from different meshes of pith at 25–100 °C. The results showed that the use of 100 mesh pith soaked in 8% (w/v) NaOH solution at 100 °C resulted in 32.47% ± 0.01% solid recovery. Follow-up fermentation with 3% (v/v) acid and 6.5% (w/v) alkali at 50 °C for enzymolysis was performed with the optimal enzyme ratio. An analysis of the surface topography and porosity before and after pretreatment showed that both the pore size of the pith and the amount of exposed cellulose increased as the mesh size increased. Interestingly, various compounds, including 42 compounds previously known to be present and 13 compounds not previously known to be present, were detected in the pretreatment liquid, while 10 types of monosaccharides, including D-glucose, D-xylose and D-arabinose, were found in the enzymatic solution. The total monosaccharide content of the pith was 149.48 ± 0.3 mg/g dry matter.

**Discussion:**

An integrated technique for obtaining value-added products from sweet sorghum pith is presented in this work. Based on this technique, lignin and hemicellulose were effectively broken down, amorphous cellulose was obtained and all sugars in the sweet sorghum pith were hydrolysed into monosaccharides. A total of 42 compounds previously found in these materials, including alcohol, ester, acid, alkene, aldehyde ketone, alkene, phenolic and benzene ring compounds, were detected in the pretreatment pith. In addition, several compounds that had not been previously observed in these materials were found in the pretreatment solution. These findings will improve the transformation of lignocellulosic biomass into sugar to create a high-value-added coproduct during the integrated process and to maximize the potential utilization of agricultural waste in current biorefinery processing.

## Introduction

The definition of waste is an unusable or unwanted substance, including material in solid, liquid or gaseous form (Sasikumar & Krishna, 2009). Straw is a type of solid waste that arises from agricultural activities and was commonly treated by open burning (Danutawat & Nguyen, 2007). However, previous studies have reported that straw burning destroys soil structure and causes farmland quality to decline (Hamer, 2003). The microorganisms on the surface are killed by straw incineration, the humus and organic matter are mineralized, the balance of the biomass system is destroyed, the physical properties of the soil are changed and soil compaction is aggravated, which affects crop growth (Johansson et al., 2014). Furthermore, the incineration of straw increases atmospheric pollutants, including sulphur dioxide, nitrogen dioxide and respirable particulate matter (PM), which is not conducive to human health (Ryan et al., 2017). When the concentration of inhalable particles reaches a certain level, the mucous membranes in the human eye, nose and throat are irritated, and a light cough, chest tightness and tearing develop (Lopez-Ponnada et al., 2017). If the smoke emitted by straw burning is combined with other pollutants, the resulting pollution can increase the fine PM 2.5 concentration and create smoggy weather, which may increase the number of sick elderly adults and children (Sabbas et al., 2003).

In the 20th century, research on the treatment and utilization of solid waste progressed, and engineering techniques employed biochemical conversion to manufacture high-value-added agricultural products from agricultural waste. The ultimate aim of biochemical conversion was to use value-added products to recycle resources (Li et al., 2010). All parts of sweet sorghum are considered an economical cereal crop for agricultural development (Reis et al., 2016; Mishra et al., 2017a). The solid waste root in sweet sorghum stalks, which is crushed to remove juice, is called sweet sorghum bagasse (SSB) and consists of a dermal layer and pith (Maureen, Isabel & Renee, 2017). Due to its high biomass content, SSB is considered a renewable fuel source and can be further biochemically converted to ethanol or butanol without reducing the fuel value (Umagiliyage et al., 2015; Cheng et al., 2008). In comparison with other crop bagasse, SSB has a favourable fuel value due to its high carbon content (Ragauskas et al., 2014; Maureen et al., 2018). Previous articles have reported using SSB for the release of structural sugars (Li et al., 2013; Wen et al., 2018). However, bagasse contains approximately 35% pith, which has a depithing efficiency of 60% (Lu et al., 2013). The pith roots in SSB have a storage function during the growth of sweet sorghum. However, after separation, SSB still contains biomass that can be converted into valuable products via effective technologies that enhance resource utilization for economic development and environmental sustainability (Shen et al., 2013). Unexpectedly, the pith roots in bagasse are primarily composed of cellulose, hemicellulose and lignin, similar to other lignocellulosic materials (Pronyk, Mazza & Tamaki, 2011). Thus, fractionation of these three primary components is necessary to release the sugar to become biofuels or coproducts (Del Río et al., 2012; Wen et al., 2013).

In recent studies, this pretreatment technology has been combined with physical and chemical processes to obtain the three primary components (Jorgensen, Haglind & Clausen, 2014; Nadeem, Dinesh & Arun, 2017; Paulien et al., 2010; Hu & Wen, 2008). Pretreatment with physical methods does not cause changes in the lignocellulosic components, but it can destroy the structure of the lignocellulosic materials, thereby increasing the efficiency of subsequent enzymatic treatment (Ballesteros et al., 2002; Baugh & McCarty, 1988; Jia et al., 2013). Pretreatment with chemical methods primarily degrades or removes lignocellulosic components, including lignin and hemicellulose, to enhance enzymatic hydrolysis (Wu et al., 2011; Arosha et al., 2015). Therefore, the combination of physical and chemical processes is conducive to the disintegration of lignocellulosic materials and improvement of the hydrolysis efficiency. Singh et al. (2017) studied the effectiveness of hydrothermal and microwave-assisted alkali pretreatment for the fractionation of areca nut husk. These researchers observed that the optimal conditions released 69.7% of the lignin, 83.5% of the hemicellulose and 69.2% of the cellulose. Recently, Wang et al. (2015) showed that thermochemical pretreatment and microbial fermentation hydrolysis of corn straw degraded less than 52.01% of the lignin and 45.7% of the hemicellulose. Furthermore, the cellulose content was increased to 59.92%. Akhtar et al. (2016) reported the effectiveness of microwave-alkali-acid pretreatment of rice straw, with degradation of 50.66% of the lignin and 39.21% of the hemicellulose and an increase of 60.07% for cellulose. Mishra et al. (2017b) reported the fungal pretreatment of SSB for lignin degradation by using synergistic CuSO_4_-syringic acid supplements, and they obtained a maximum lignin degradation of 35.9% ± 1.3%. Heredia-Olea, Pérez-Carrillo & Serna-Saldívar (2012) showed using response surface methodology that different acid hydrolyses affected the conversion of SSB into C5 and C6 sugars and yeast inhibitors, and they liberated 56–57% of the total sugars in SSB.

Huang, Kuan & Chang (2018) reported that particle size has an effect on the microwave pyrolysis of corn stover, but no studies have investigated the effect of particle size on the hydrolysis of sweet sorghum pith. However, these types of studies on the hydrolysis of SSB have been reported (Sipos et al., 2009; Zhang et al., 2011). A combination of chemical and physical processes was used to hydrolyse the resistant structure of the pith in this study. The effects of particle size, alkali and acid concentrations, temperature and enzyme dose on the production of sugar and unknown products were investigated in the integrated process. The objective of this study was to thoroughly analyse and compare the effects of particle size on the value-added products of pith under various hydrolysis conditions from a physicochemical and microscopic perspective. Thus, we hope to provide data to support the reasonable use of natural resources, which may be beneficial to solving increasingly serious environmental problems and promote environmental protection and the recycling of other compounds.

## Materials and Methods

### Materials

Sweet sorghum bagasse was dried at a constant temperature in a drying oven at 60 °C for 24 h, and the pith was then separated from the SSB. The dried pith was crushed into small pieces using a three-roller mill (Ampro Sugar Cane Mill), and different particle sizes of pith were collected. The particle sizes are referred to as 10–20, 50 and 100 mesh in this study.

### Alkali and acid pretreatment

Unpretreated pith was utilized as starting material for fractionation. First, 0.2 g of pith of different meshes (10–20, 50 and 100 mesh) was treated with different concentrations of NaOH (5%, 6.5% and 8%, w/v) and HCl (1.5%, 3% and 4.5%, v/v) in aqueous solution at different temperatures (25, 50, 75 and 100 °C) for 2 h at a solid to liquid ratio of 1:20 (g/mL). To increase the contact between the pretreatment solution and the samples, 200 μL of 1% (v/v) Tween-80 was added. Subsequently, each mixed solution was centrifuged at 6,000 rpm for 15 min, and the supernatant was analysed using gas chromatography-quadrupole mass spectrometry (GC-MS). The collected solid fractions were neutralized to pH 6.5−7.5 with deionized water and further filtered with a vacuum pump. The filtered solid biomass was then dried in a drying oven at 60 °C until its weight was constant, and the weight was then recorded.

### Enzymatic hydrolysis

Pith (0.1 g) pretreated under different conditions was then treated with commercial cellulase® (activity 3,000 IU/g) and β-glucosidase® (activity 100 IU/g) in 50 mL flasks provided by Solarbio (Beijing, China). Pretreated pith samples were added to 0.05M sodium citrate buffer (pH 4.8) to a concentration of 10 g/L, and the resulting mixture was then mixed with 10 mL of deionized water. To these flasks, five different concentrations of enzymes were added (cellulase and β-glucosidase at concentrations of 10 and 20, 15 and 25, 20 and 30, 25 and 35, and 30 and 40 IU/(g dry biomass), respectively). Each concentration was tested in triplicate. Hydrolysis was performed for 72 h (Li et al., 2013) in a shaking incubator at 50 °C and 150 rpm (Wu et al., 2011; Zheng et al., 2018). Afterwards, the hydrolysates were centrifuged (10,000 rpm, 10 min) (Singh et al., 2017; Li et al., 2016), and the liquid fraction was collected for further experiments (Yan et al., 2015).

### Analysis procedures

#### Solid recovery

The recovery rate of the pith was estimated using Eq. (1):

(1) $${\rm{Recovery\ }}\left(\% \right){\rm{ }} = {\rm{ }}\left({{M_1}{\rm{ }}/{\rm{ }}{M_0}} \right){\rm{ }} \times {\rm{ }}100\% $$

where *M*_1_ is the mass of the pretreated dry solid (g) and *M*_0_ is the mass of the raw materials (g).

#### Emission scanning electron microscopy analysis

The morphological characteristics of the untreated and pretreated pith were observed using cold field emission scanning electron microscopy (SEM) (Model: JSM-6701F, Hitachi Electronics Co., Ltd., Tokyo, Japan). Prior to drying, the pith was placed on conductive tape and plated separately. The images were obtained with SEM at magnifications of 500–2,000 by using an accelerating voltage of 0.5–30 kV. Porosity was determined using the Barrett–Joyner–Halenda (BJH)/Dollimore–Heal (DH) method. Outgas temp: 120.0 °C, analysis gas: nitrogen (Yan et al., 2015).

#### Total sugar detection

The total sugar content was determined using the phenol-sulphuric acid method, where glucose is the standard solution used to develop a standard curve and the corresponding regression equation. Hydrolysates were mixed with one mL of phenol solution and five mL of concentrated sulphuric acid. The absorbance was determined at 490 nm with the zero tube as the reference at room temperature for 30 min. Then, a regression equation was used to calculate the total sugar concentration.

#### Monosaccharide analysis of the pretreatment solution

The associated monosaccharides in the hydrolysate were analysed using high-performance liquid chromatography (HPLC) (Agilent 1200 Infinity; Agilent Technologies, Santa Clara, CA, USA). D-Arabinose, L-fucose, D-galactose, D-galacturonic acid, D-glucose, D-gluconic acid, D-mannose, D-ribose, L-rhamnose and D-xylose were used as sugar standards for the HPLC analysis. The hydrolysates were derivatized with the reagent 1-phenyl-3-methyl-pyrazolone and then determined using a Thermo C18 (4.6 × 250 mm, 5 μm) column at 25 °C with an eluent (0.1 mol/L, pH 7.0, phosphate-buffered solution:acetonitrile = 82:18 (v/v)) at a flow rate of 1.0 mL/min.

#### Linear retention index calculation and data analysis

The linear retention index (LRI) was calculated according to Eq. (3) by using a homologous reference series of alkanes and saturated fatty acid methyl esters consisting of standards ranging from C8 to C35 that were purchased from Millipore Sigma (Burlington, MA, USA). A standard mixture of the compounds was prepared at a concentration of 1,000 mg/L. The LRI database was built by using CromatoPlus Spectra software (Chromaleont, Messina, Italy), which was also able to extrapolate LRI values for TGs eluted earlier than C8. Then, analyses were performed by using CromatoPlus Spectra software, which directly matched the LRI automatically calculated for each peak with the previously created LRI database.

Dool & Kratz (1963) extended the applicability of retention indices to temperature-programmed gas chromatography analyses, utilizing retention times instead of their logarithm and thus defining the LRI as shown in Eq. (2):

(2) $${\rm{LRI}} = 100\left({z + {{{t_{Ri}}-{t_{Rz}}} \over {{t_{R\left({Z + 1} \right)}}-{t_{Rz}}}}} \right)$$

Within this context, a generalized form for the LRI is shown in Eq. (3):

(3) $${\rm{LRI}} = 100 \cdot \left(n \right) + 100 \cdot \left({m-n} \right) \cdot {{{t_{ri}}-{t_{rn}}} \over {{t_{rm}}-{t_{rn}}}}$$

where

- LRI = Linear retention index of ‘*i*’
- *i* = Unknown compounds in the pretreatment solution that is being analysed
- *n* = Carbon number of the alkane that elutes before ‘*i*’
- *m* = Carbon number of the alkane that elutes after ‘*i*’
- *t_ri_* = Retention time of ‘*i*’
- *t_rn_* = Retention time of the alkane that elutes before ‘*i*’
- *t_rm_* = Retention time of the alkane that elutes after ‘*i*’

#### Analysis of the unknown compounds in the pretreatment solution

A GC-MS (Agilent 6890-78; Agilent Technologies, Santa Clara, CA, USA) equipped with a DB-5 MS column (30 m × 0.25 mm × 0.25 μm) and flame ionization detector was used to analyse the obtained compounds. The ion source temperature and quadrupole temperature were 230 and 150 °C, respectively. High-purity He gas was injected into the chromatograph as the carrier gas at a flow rate of 1.0 mL/min. For the experiment, two μL samples were injected into the chromatograph by the pulseless split injection mode at a flow rate of 50 mL/min, pulse pressure of 50 psi, and split time of 0.75 min (scanning unit: 50–500 (Da), scan time: 0.3 s). The temperature was first set to 150 °C for 1 min, then increased to 230 °C at 10 °C/min, and finally increased to 300 °C at 40 °C/min and maintained for 2 min. The analysis time was 20 min.

### Statistical analysis

All data represent the results of three independent samples (flasks). The error bars indicate the standard deviation from the mean of experiments performed in triplicate. The data were analysed using one-way analysis of variance followed by the Student–Newman–Keuls test. A two-tailed *P*-value < 0.05 was considered to indicate statistically significant differences. SPSS 18.0 software for Windows was used for the statistical analyses (SPSS Inc., Chicago, IL, USA).

## Results

### Surface topography and porosity of pith, untreated or pretreated with acid or alkali

**Figure 1:**
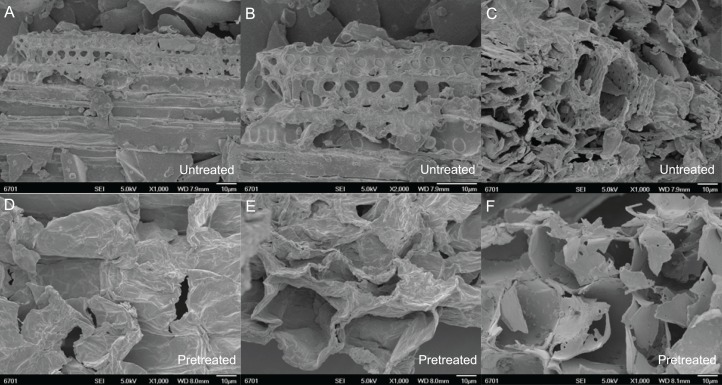
Microscopic performance of mechanical smashing and chemical catalysis of untreated and pretreated pith root in SSB. (A–C) Untreated 10–20, 50 and 100 mesh pith at the microscopic level. (D–F) Pretreated 10–20, 50 and 100 mesh pith with the best microscopic examples in this study.

The pore size of pith pretreated with different concentrations of acid and alkali agents was studied using the BJH/DH method. The effects of pretreatment acid and alkali concentrations on the pore size of the pith are shown in Table 1. The pore size of the pith increased with increasing mesh size before and after pretreatment, and the pore size of the pretreated pith was much higher than that of the untreated pith. The structure of the pith was thus loosened both before and after pretreatment, and the degree of structural looseness increased with increasing mechanical cutting strength (Fig. 1). Figure 1 shows a microscopic analysis of the untreated and pretreated pith roots in SSB under mechanical smashing and chemical catalysis. The structural porosity was positively correlated with the cutting strength before pretreatment. The size of the surface pores of the untreated pith was only 3.20 ± 0.03 nm at 10–20 mesh, while the size of the pores at 50 mesh and 100 mesh was 7.19 ± 0.01 nm and 9.50 ± 0.05 nm, respectively.

**Table 1:** Effect of differing concentrations of acid or alkali pretreatment on the pore size.

Pore diameter *Dv* (*d*) (*nm*)							
Parameter	Untreated	1.5% acid	3% acid	4.5% acid	5% alkali	6.5% alkali	8% alkali
10–20 mesh	3.20 ± 0.03	7.60 ± 0.02	11.99 ± 0.05	12.12 ± 0.02	11.92 ± 0.01	12.92 ± 0.01	13.11 ± 0.03
50 mesh	7.19 ± 0.01	9.78 ± 0.04	12.22 ± 0.03	12.34 ± 0.01	19.23 ± 0.04	23.22 ± 0.03	23.59 ± 0.01
100 mesh	9.50 ± 0.05	10.86 ± 0.01	12.35 ± 0.02	12.46 ± 0.05	21.27 ± 0.03	23.98 ± 0.05	24.05 ± 0.04

As shown in Fig. 1, there are more pores in the pith treated with the 100 mesh than in that treated with the 10–20 and 50 meshes. Furthermore, the pore size in the pith fragments treated with the 100 mesh was larger than that in the fragments treated with the 10–20 and 50 meshes. This difference is correlated with an increase in cutting intensity. As seen in Table 1, the pore size of the pith increased as the acid and alkali concentrations increased. The pore sizes of 10–20, 50 and 100 mesh pith pretreated with 3% (v/v) acid were 11.99 ± 0.05 nm, 12.22 ± 0.03 nm and 12.35 ± 0.02 nm, respectively, which were approximately 57.8%, 24.95% and 13.72% greater than that of pith pretreated with 1.5% (v/v) acid. The pore sizes of 10–20, 50 and 100 mesh pith pretreated with 4.5% (v/v) acid were approximately 1.08%, 1.01% and 0.89% greater than that of pith pretreated with 3% (v/v) acid, respectively. The pore sizes of 10–20, 50 and 100 mesh pith pretreated with 6.5% (w/v) alkali were 12.92 ± 0.01 nm, 23.22 ± 0.03 nm and 23.98 ± 0.05 nm, respectively, which was approximately 8.4%, 20.7% and 12.7% greater than that of pith pretreated with 5% (w/v) alkali (Table 1). However, the pore size of 10–20, 50 and 100 mesh pith pretreated with 8% (w/v) alkali was approximately 1.5%, 1.5% and 0.3% greater than that of pith pretreated with 6.5% (w/v) alkali. There was only a slight difference between the pore sizes of pith pretreated with 3% (v/v) acid and 4.5% (v/v) acid, similar to the difference observed between 6.5% (w/v) alkali and 8% (w/v) alkali. This result suggested that 3% (v/v) acid and 6.5% (w/v) alkali may be more appropriate than 4.5% (v/v) acid and 8% (w/v) alkali for treating pith due to environmental protection and economic savings.

### Solid recovery of pith with different pretreatment conditions

**Figure 2:**
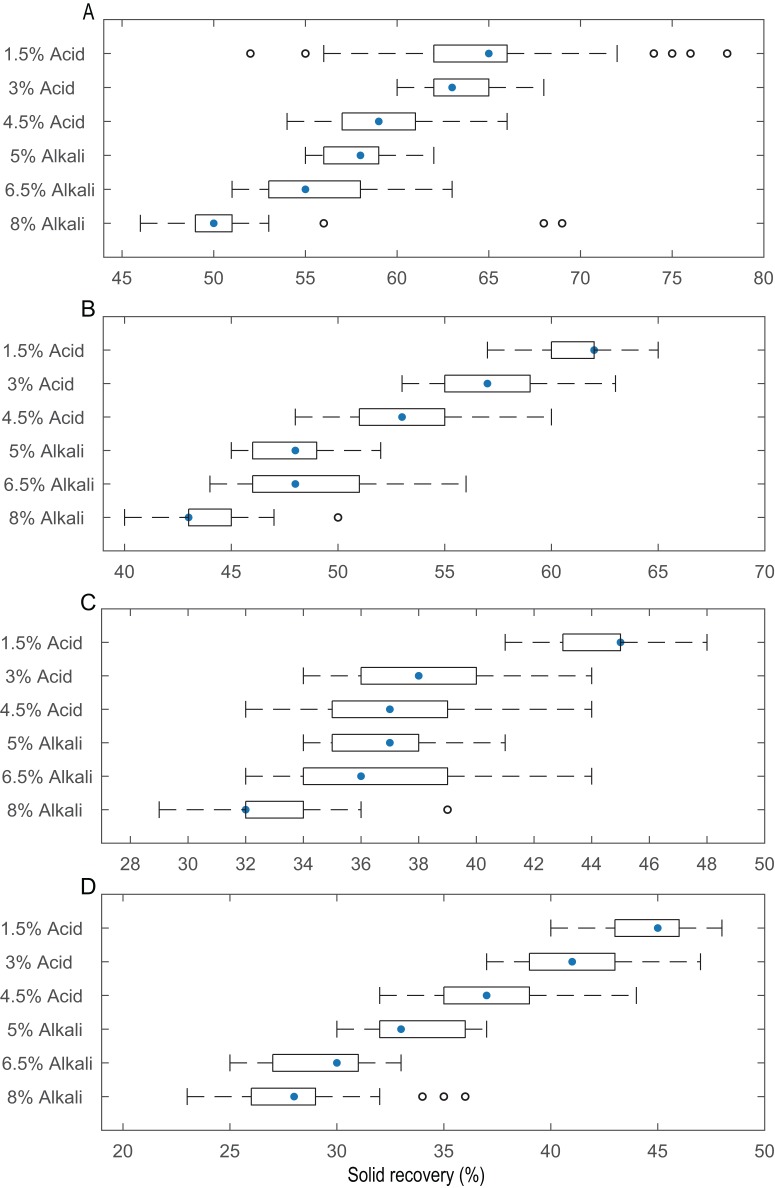
Solid yield of the pith after pretreatment with different concentrations of acid and alkali at different temperatures. (A) Box plot of an included set of data at 25 °C. (B) Box plot of an included set of data at 50 °C. (C) Box plot of an included set of data at 75 °C. (D) Box plot of an included set of data at 100 °C.

The effects of temperature (25, 50, 75 and 100 °C), acid (1.5%, 3% and 4.5%, v/v) and alkali (5%, 6.5% and 8%, w/v) treatments for 2 h on the solid recovery of 10–20, 50 and 100 mesh pith are shown in Table 2. After 1.5% (v/v) acid pretreatment, the yields of the residues varied from 88.05% ± 0.03% to 39.88% ± 0.03% with increasing pretreatment temperature (from 25 to 100 °C) and mesh size (10–20 to 100 mesh). After the pith was further treated with 4.5% (v/v) acid, the yields of the remaining residues were dramatically reduced to 83.22% ± 0.04%–38.11% ± 0.02%. After 5% (w/v) alkali pretreatment, the yields of the residues varied from 65.70% ± 0.03% to 36.89% ± 0.02% with increasing pretreatment temperature (from 25 to 100 °C) and mesh size (10–20 to 100). After the pith was further treated with 8% (w/v) alkali, the yields of the remaining residues notably decreased to 60.35% ± 0.05%–32.47% ± 0.01%. Analysis of the pretreated pith residues showed that the alkali treatment is better than the acid treatment. There is no great difference in the solid recoveries of the pith with 3% (v/v) acid and 4.5% (v/v) acid, similar to the difference between 6.5% (w/v) alkali and 8% (w/v) alkali. This finding again showed that 3% (v/v) acid and 6.5% (w/v) alkali were more suitable than 4.5% acid (v/v) and 8% (w/v) alkali for this process. A box plot (Fig. 2) of the solid recovery rate was generated using R and C^+^ to more clearly present the solid recovery at different concentrations and temperatures by highlighting the quartile, minimum and maximum values. Solid recovery gradually decreased as the temperature and the acid and alkali concentrations increased. When the temperature was below 50 °C, the normal value distribution was narrower than when the temperature was greater than 50 °C. The solid recovery of 3% (v/v) acid-pretreated pith in Fig. 2A was approximately 59–68%, while the solid recoveries of 3% (v/v) acid-pretreated pith in Figs. 2B–2D were approximately 53–68%, 34–44% and 36–47%, respectively. The solid recovery of 6.5% (w/v) alkali-pretreated pith in Fig. 2A was approximately 51–63%, while the solid recoveries of 6.5% (w/v) alkali-pretreated pith in Figs. 2B–2D were approximately 43–56%, 32–44% and 25–33%, respectively. Some unusual points emerged regarding the 8% (w/v) alkali treatment at every temperature. For example, when the temperature was 25 or 100 °C, these unusual points were more extreme than those at 50 or 75 °C. This result showed that excessive temperature and acid/alkali concentration may be detrimental to the degradation of lignocellulosic biomass.

**Table 2:** Solid recovery of different particle sizes of pith for various pretreatment conditions.

Concentration	Temperature (°C)	Solid recovery (%)		
		10–20 mesh	50 mesh	100 mesh
1.5% acid	25	88.05 ± 0.03	82.25 ± 0.02	74.43 ± 0.04
	50	86.1 ± 0.01	73.05 ± 0.05	59.1 ± 0.05
	75	67.52 ± 0.03	55.43 ± 0.02	49.38 ± 0.02
	100	60.31 ± 0.04	46.85 ± 0.03	39.88 ± 0.03
3% acid	25	84.31 ± 0.03	72.78 ± 0.04	58.77 ± 0.06
	50	82.71 ± 0.02	70.13 ± 0.04	56.11 ± 0.03
	75	66.15 ± 0.05	52.34 ± 0.06	47.23 ± 0.01
	100	59.30 ± 0.03	46.25 ± 0.01	38.47 ± 0.05
4.5% acid	25	83.22 ± 0.04	71.44 ± 0.05	57.12 ± 0.02
	50	80.67 ± 0.06	68.55 ± 0.03	54.33 ± 0.03
	75	63.45 ± 0.03	50.23 ± 0.01	46.66 ± 0.04
	100	57.34 ± 0.01	44.18 ± 0.05	38.11 ± 0.02
5% alkali	25	65.70 ± 0.03	64.35 ± 0.01	60.15 ± 0.04
	50	62.45 ± 0.02	60.38 ± 0.03	57.25 ± 0.01
	75	60.14 ± 0.05	50.21 ± 0.04	46.34 ± 0.03
	100	54.76 ± 0.03	40.36 ± 0.02	36.89 ± 0.02
6.5% alkali	25	64.35 ± 0.04	60.65 ± 0.03	50.65 ± 0.01
	50	62.11 ± 0.01	58.32 ± 0.05	48.28 ± 0.06
	75	59.15 ± 0.02	49.95 ± 0.06	45.23 ± 0.03
	100	53.37 ± 0.03	38.25 ± 0.01	34.47 ± 0.05
8% alkali	25	60.35 ± 0.05	59.01 ± 0.03	48.19 ± 0.02
	50	59.34 ± 0.04	58.01 ± 0.02	47.63 ± 0.04
	75	56.15 ± 0.06	47.97 ± 0.01	43.23 ± 0.02
	100	51.22 ± 0.02	36.25 ± 0.03	32.47 ± 0.01

### Total sugar release of pith for different enzyme ratios

As mentioned previously, the enzyme dose was optimized to maximize sugar release at 50 °C, 6.5% (w/v) alkali, and 3% (v/v) acid. The saccharification of the pith was implemented in a shaking incubator at 50 °C and 150 rpm for 72 h using five different enzyme ratios. As shown in Table 3, after acid pretreatment and alkali pretreatment, for 10 U/g cellulase and 20 U/g β-glucosidase, the production of total sugar increased from 0.79 ± 0.03 mg/mL cellulase and 1.79 ± 0.03 mg/mL β-glucosidase for 10–20 mesh pith to 1.08 ± 0.05 mg/mL and 2.47 ± 0.07 mg/mL, respectively, for the 100 and 50 mesh pith. The total sugar greatly increased with the increase from 10 U/g cellulase and 20 U/g β-glucosidase to 20 U/g cellulase and 30 U/g β-glucosidase. For pith hydrolysed with 20 U/g cellulase and 30 U/g β-glucosidase, the sugars increased from 0.81 ± 0.02 mg/mL and 2.34 ± 0.02 mg/mL for 10–20 mesh pith to 1.23 ± 0.04 mg/mL and 2.88 ± 0.04 mg/mL for 100 and 50 mesh pith. The total sugar increased slightly with the increase from 20 U/g cellulase and 30 U/g β-glucosidase to 30 U/g cellulase and 40 U/g β-glucosidase. The maximum total sugar content was 2.88 ± 0.04 mg/mL, which occurred with 0.1 g of 50 mesh alkali-pretreated pith, 20 U/g cellulase and 30 U/g β-glucosidase.

**Table 3:** Total sugar content of the different particle sizes of pith for different hydrolysed enzyme ratios.

Enzyme dose (U/g, cellulase and β-glucosidase)	Total sugar (mg/mL)					
	Acid (10–20 mesh)	Acid (50 mesh)	Acid (100 mesh)	Alkali (10–20 mesh)	Alkali (50 mesh)	Alkali (100 mesh)
10 and 20	0.79 ± 0.03	0.97 ± 0.01	1.08 ± 0.05	1.79 ± 0.03	2.47 ± 0.07	2.23 ± 0.03
15 and 25	0.79 ± 0.02	1.01 ± 0.02	1.15 ± 0.02	1.92 ± 0.01	2.56 ± 0.08	2.39 ± 0.06
20 and 30	0.81 ± 0.02	1.03 ± 0.01	1.23 ± 0.04	2.34 ± 0.02	2.88 ± 0.04	2.48 ± 0.05
25 and 35	0.88 ± 0.01	1.09 ± 0.04	1.27 ± 0.02	2.32 ± 0.01	2.81 ± 0.05	2.49 ± 0.06
30 and 40	0.86 ± 0.03	1.05 ± 0.05	1.22 ± 0.03	2.33 ± 0.02	2.83 ± 0.01	2.46 ± 0.04

**Figure 3:**
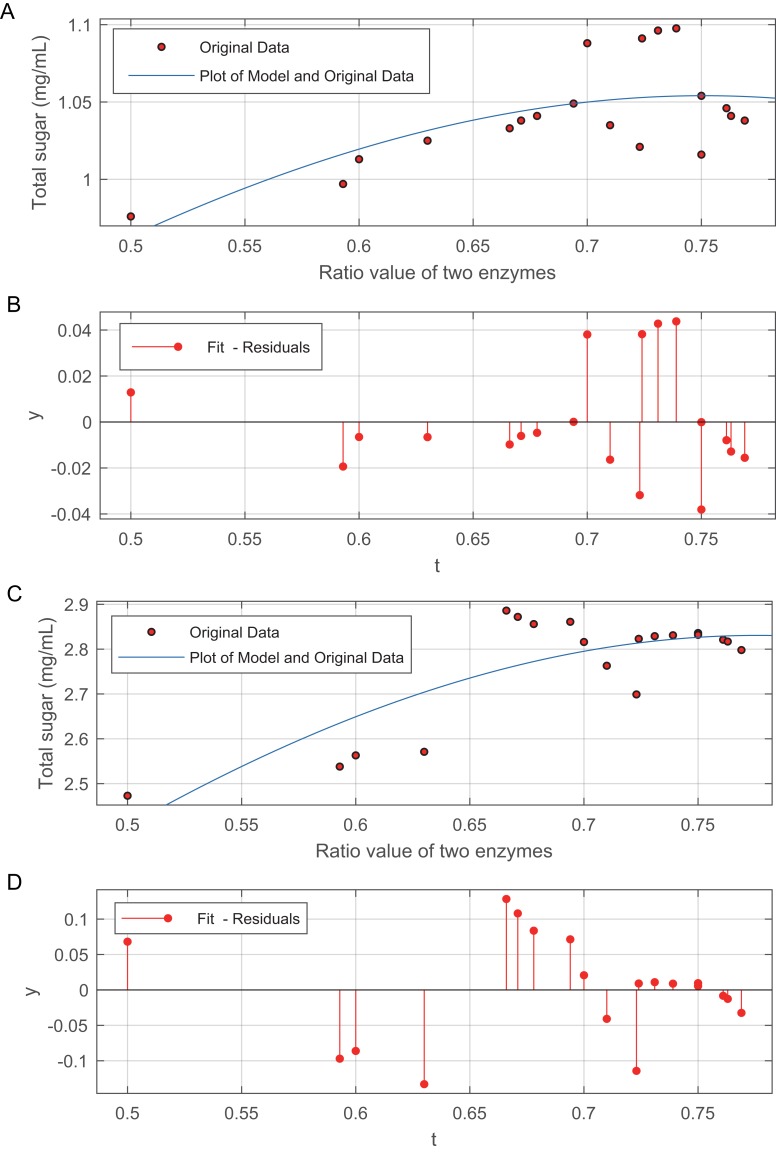
Effect of the ratio of cellulase to β-glucosidase on total sugar release. (A) Plot of the model and original data for 50 mesh pith treated at 50 °C and with 3% (v/v) acid. (B) The method of weighted residuals was used to analyse the model of the data from the total sugar quantification. (C) Plot of the model and original data for 50 mesh pith treated at 50°C and with 6.5% (w/v). (D) The method of weighted residuals was used to analyse the model of the data from the total sugar quantification.

The relationship between the two enzyme ratios and the total sugar is shown in Fig. 3. The data in Fig. 3B are modelled by Eq. (4):

(4) $$f\left(x \right) = {\rm{ }}-1.324{x^2} + 2.018x + 0.2859$$

and Fig. 3C. The data in Fig. 3D are modelled by Eq. (5):

(5) $$f\left(x \right) = {\rm{ }}-4.993{x^2} + 7.922x +-0.3055$$

The method of weighted residuals was used to analyse the model of the data from the total sugar. Figures 3A and 3C show that, the total sugar content sharply increased when the ratio between the two enzymes ranged from 0.5 to 0.67. The increases in total sugar content gradually levelled off, regardless of acid or alkali treatment, when the ratio of the two enzymes was greater than 0.67.

### Detection of monosaccharide release by HPLC

**Figure 4:**
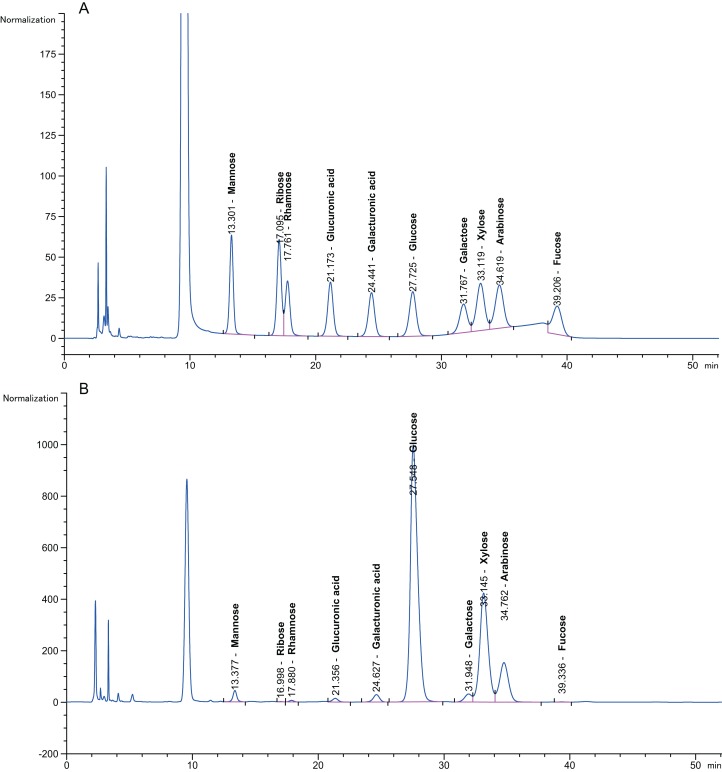
Chromatogram of monosaccharides. (A) Chromatogram of monosaccharide standard solution. (B) Chromatogram of 10 monosaccharides in the hydrolysis solution. The peak order of monosaccharides is 1, mannose; 2, ribose; 3, rhamnose; 4, gluconic acid; 5, galacturonic acid; 6, glucose; 7, galactose; 8, xylose; 9, arabinose; and 10, fucose. Separation conditions: detection at 245 nm with a Thermo C18 column at 25 °C, injection volume of 10 μL at 25 °C, eluent of 0.1 mol/L pH 7.0 phosphate-buffered solution: acetonitrile = 82:18 (v/v), and a flow rate of 1.0 mL/min.

Pretreatment with 3% (v/v) acid and 6.5% (w/v) alkali was performed to first depolymerize the hemicellulose and lignin at a low temperature (50 °C, 72 h) to avoid excessive loss of effective components at high temperature (100 °C); then, the sugars were hydrolysed with enzymes at their optimal ratio, which produced monosaccharide that was detected using HPLC. Figure 4A presents a chromatogram of a monosaccharide standard solution containing D-arabinose, L-fucose, D-galactose, D-galacturonic acid, D-glucose, D-gluconic acid, D-mannose, D-ribose, L-rhamnose and D-xylose. Figure 4B shows a chromatogram of the 10 monosaccharides in the hydrolysates for the optimized separation conditions. The peak separation was excellent and allowed the identification and quantification of all neutral sugars of interest. Rovio et al. (2008) reported that the monosaccharide composition in plant fibre materials determined by capillary zone electrophoresis included six neutral carbohydrates. This experiment simultaneously separated 10 neutral carbohydrates.

The content of the 10 neutral monosaccharides in the hydrolysate is described in Table 4. Variation in the monosaccharide content for the different particle sizes of the pith was evident throughout the data set. There were relatively high concentrations of glucose, xylose and arabinose (2.84 ± 0.01–68.23 ± 0.04 mg/g dry matter, 11.95 ± 0.05–72.76 ± 0.07 mg/g dry matter and 2.28 ± 0.04–25.93 ± 0.05 mg/g dry matter, respectively). Fucose, ribose and rhamnose (0.15 ± 0.03–0.89 ± 0.01 mg/g, 0.12 ± 0.02–0.33 ± 0.06 mg/g and 0.06 ± 0.07–0.46 ± 0.06 mg/g, respectively) were regarded as minor lignocellulosic sugar components. Li et al. (2016) reported the hydrolysis of two-stage (alkali-acid)-pretreated SSB with more sugar obtained (246.34 mg/g) than in this study (149.48 ± 0.3 mg/g dry matter).

**Table 4:** Neutral monosaccharide content in enzymatic hydrolysate of pith for different pretreatment methods, and 20 U/g of cellulase, 30 U/g of β-glucosidase hydrolysed.

Pretreatment method	Particle size (mesh)	Monosaccharide (mg/g dry matter)									
		Ara	Fuc	Gal^a^	Gal^b^	Glu^a^	Glu^b^	Man	Rib	Rha	Xyl
Acid	10–20	6.48 ± 0.01	0.15 ± 0.03	4.32 ± 0.07	0.98 ± 0.06	47.07 ± 0.05	0.94 ± 0.03	2.54 ± 0.03	0.19 ± 0.05	0.21 ± 0.01	17.10 ± 0.06
	50	6.60 ± 0.03	0.17 ± 0.01	5.70 ± 0.02	1.05 ± 0.01	50.12 ± 0.03	1.95 ± 0.05	3.22 ± 0.06	0.23 ± 0.04	0.27 ± 0.04	15.51 ± 0.04
	100	25.93 ± 0.05	0.72 ± 0.02	7.49 ± 0.03	5.18 ± 0.02	68.23 ± 0.04	2.02 ± 0.02	3.97 ± 0.07	0.26 ± 0.07	0.32 ± 0.02	57.83 ± 0.01
Alkali	10–20	2.28 ± 0.04	0.54 ± 0.04	0.70 ± 0.05	0.33 ± 0.04	2.84 ± 0.01	1.30 ± 0.04	0.23 ± 0.05	0.13 ± 0.01	0.06 ± 0.07	11.95 ± 0.05
	50	14.72 ± 0.05	0.89 ± 0.01	2.27 ± 0.03	1.40 ± 0.01	52.85 ± 0.07	2.53 ± 0.01	1.27 ± 0.01	0.33 ± 0.06	0.46 ± 0.06	72.76 ± 0.07
	100	10.22 ± 0.07	0.70 ± 0.02	2.07 ± 0.06	1.94 ± 0.02	11.98 ± 0.05	2.73 ± 0.02	0.79 ± 0.03	0.12 ± 0.02	0.13 ± 0.02	25.39 ± 0.2

**Note:**

Ara, arabinose; Fuc, fucose; Gal^a^, galactose; Gal^b^, galacturonic acid; Glu^a^, glucose; Glu^b^, gluconic acid; Man, mannose; Rib, ribose; Rha, rhamnose; Xyl, xylose.

### Analysis of unknown compounds in the pretreatment solution using GC-MS

An integrated process was used to treat the pith, and GC-MS was used for the identification of specific compounds in the pretreatment solution. The GC-MS instrument used in this study was equipped with a DB-5 MS column (30 m × 0.25 mm × 0.25 μm) and an RI detector and used an ion source temperature of 230 °C. As shown in Table 5, GC/MS and the LRI were used to identify the compounds already known in the pretreatment solution, and a total of 42 components were detected. The main components included three alcohol compounds, 15 ester compounds, six acid compounds, four alkene compounds, five aldehyde ketone compounds, five phenolic compounds and four benzene ring compounds.

**Table 5:** Analysis of the compounds already known to be present in the pretreatment solution, especially alcohol, ester, acid, aldehyde ketone, alkene, phenolic and benzene ring compounds, by GC/MS and the linear retention index.

Type of compounds	CAS name	Retention time (min)	IUPAC standard InChIKey	CAS registry number	Molecular weight	Molecular formula	Stereoisomers	Similarity %	Linear retention index (LRI)	Linear retention index of literature
Alcohol compounds	1-Butanol, 3-methyl-	3.36	PHTQWCKDNZKARW-UHFFFAOYSA-N	123-51-3	88.1482	C_5_H_12_O	–	92	739	727 (Haken & Korhonen, 1985)
	α-Terpineol	13.03	WUOACPNHFRMFPN-SECBINFHSA-N	98-55-5	154.2493	C_10_H_18_O	L-α-TerpineolTerpineolcis-α-terpineol	89	1,221	1,190 (Hazzit et al., 2006)
	Cedrol	26.53	SVURIXNDRWRAFU-WINGCZCQSA-N	77-53-2	222.3663	C_15_H_26_O	IsocedrolIsocedranolFunebrolEpicedrol	88	1,598	1,596 (Adams & Nguyen, 2005a)
Ester compounds	Ethyl acetate	2.85	XEKOWRVHYACXOJ-UHFFFAOYSA-N	141-78-6	88.1051	C_4_H_8_O_2_	–	92	611	600 (Takeoka, Buttery & Flath, 1992)
	Propanoic acid, TMS derivative	3.12	QVSRWXFOZLIWJS-UHFFFAOYSA-N	16844-98-7	146.2597	C_6_H_14_O_2_Si	–	94	800	796.2 (Tret’yakov, 2008)
	Ethyl 6-bromohexanoate	19.15	DXBULVYHTICWKT-UHFFFAOYSA-N	25542-62-5	223.107	C_8_H_15_BrO_2_	–	93	1,221	1,282 (Komárek, Hornová & Churácek, 1982)
	Benzoic acid, ethyl ester	12.31	MTZQAGJQAFMTAQ-UHFFFAOYSA-N	93-89-0	150.1745	C_9_H_10_O_2_	–	95	–	–
	Octanoic acid, ethyl ester	12.86	YYZUSRORWSJGET-UHFFFAOYSA-N	106-32-1	172.2646	C_10_H_20_O_2_	–	96	1,182	1,184 (Takeoka, Buttery & Flath, 1992)
	Silicic acid, diethyl bis(trimethylsilyl) ester	38.96	QZGNEODXJZDIBK-UHFFFAOYSA-N	3555-45-1	296.585	C_10_H_28_O_4_Si_3_	–	98	–	–
	Nonanoic acid, ethyl ester	12.83	BYEVBITUADOIGY-UHFFFAOYSA-N	123-29-5	186.2912	C_11_H_22_O_2_	–	98	1,281	1,279 (Takeoka, Buttery & Flath, 1992)
	Decanoic acid, ethyl ester	19.41	RGXWDWUGBIJHDO-UHFFFAOYSA-N	110-38-3	200.3178	C_12_H_24_O_2_	–	96	2,453	1,406 (Cakir, 2004)
	1,5-Dimethyl-1-vinyl-4-hexenyl butyrate	9.91	FHLGUOHLUFIAAA-UHFFFAOYSA-N	78-36-4	224.3392	C_14_H_24_O_2_	–	98	1,421	1,422 (Sibanda et al., 2004)
	Dodecanoic acid, ethyl ester	25.57	MMXKVMNBHPAILY-UHFFFAOYSA-N	106-33-2	228.3709	C_14_H_28_O_2_	–	98	1,579	1,578 (Takeoka et al., 1990)
	1,2-Benzenedicarboxylic acid, bis(2-methylpropyl) ester	33.21	MGWAVDBGNNKXQV-UHFFFAOYSA-N	84-69-5	278.3435	C_16_H_22_O_4_	–	92	1,868	1,863 (Ramsey et al., 1980)
	Dibutyl phthalate	35.59	DOIRQSBPFJWKBE-UHFFFAOYSA-N	84-74-2	278.3435	C_16_H_22_O_4_		94	1,922	1,919 (Yamaguchi & Shibamoto, 1981)
	Hexadecanoic acid, methyl ester	34.51	FLIACVVOZYBSBS-UHFFFAOYSA-N	112-39-0	270.4507	C_17_H_34_O_2_	–	96	1,907	1,904.1 (Kittiratanapiboon, Jeyashoke & Krisnangkura, 1998)
	Hexadecanoic acid, ethyl ester	36.13	XIRNKXNNONJFQO-UHFFFAOYSA-N	628-97-7	284.4772	C_18_H_36_O_2_	–	97	1,978	1,978 (Takeoka, Perrino & Buttery, 1996)
	Fumaric acid, 2,6-dimethoxyphenyl 2-ethylhexyl ester	45.15	SVCXTDDDLHQROL-OUKQBFOZSA-N	–	364.4327	C_20_H_28_O_6_	–	90	2,594	2,593 (V.G. Zaikin, 2013, personal communication)
Acid compounds	3-Amino-4-methylbenzoic acid	6.09	XKFIFYROMAAUDL-UHFFFAOYSA-N	2458-12-0	151.1626	C_8_H_9_NO_2_	–	92	–	–
	1-Cyclohexene-1-carboxylic acid	17.83	NMEZJSDUZQOPFE-UHFFFAOYSA-N	636-82-8	126.1531	C_7_H_10_O_2_	–	99	–	–
	Benzoic acid, 4-pentyl-	16.17	CWYNKKGQJYAHQG-UHFFFAOYSA-N	26311-45-5	192.2542	C_12_H_16_O_2_	–	99	–	–
	Glutaric acid, hexyl 3-methylbut-2-yl ester	25.89	GNEZDIABYORDPY-UHFFFAOYSA-N	–	286.4070	C_16_H_30_O_4_	–	90		1,960 (V.G. Zaikin, 2009, personal communication)
	4-(Anisylideneamino)-cinnamic acid	3.28	UIELBEHBZKVMEI-XOCFQZNGSA-N	25959-50-6	281.3059	C_17_H_15_NO_3_	–	92	–	–
	Vanillylmandelic acid, 3TMS derivative	27.66	QGOJNGYLGCWPKN-UHFFFAOYSA-N	55268-66-1	414.7161	C_18_H_34_O_5_Si_3_	–	95	1,896	1,908 (Halket et al., 1999)
Aldehyde ketone compounds	1,4-Dibromo-6-methoxycyclohexa-1,2,4-triene	3.07	XSXQPOOLARJBOW-UHFFFAOYSA-N	–	264.924	C_7_H_5_Br_2_O	–	89	–	–
	1-(3,4-Dimethoxyphenyl)-4,4,4-trifluorobutane-1,3-dione	21.61	QYQACDYPMNIAMN-UHFFFAOYSA-N	–	276.211	C_12_H_11_F_3_O_4_	–	93	1,431	1,430 (Midgley et al., 1979)
	5,9-Undecadien-2-one, 6,10-dimethyl-, (E)-	21.38	HNZUNIKWNYHEJJ-FMIVXFBMSA-N	3796-70-1	194.3132	C_13_H_22_O	5,9-Undecadien-2-one, 6,10-dimethyl-, (Z)-5,9-Undecadien-2-one, 6,10-dimethyl-	94	1,455	1468 (Tayoub et al., 2006)
	2(1H)-Naphthalenone, octahydro-3-(1-hydroxy-1-methylethyl)-5,8a-dimethyl-	22.34	SPOVXCZOIMHFTC-UHFFFAOYSA-N	–	238.371	C_15_H_26_O_2_	–	89	–	–
	5,9,13-Pentadecatrien-2-one,6,10,14-trimethyl-	32.47	LTUMRKDLVGQMJU-UHFFFAOYSA-N	762-29-8	262.4302	C_18_H_30_O	5,9,13-Pentadecatrien-2-one, 6,10,14-trimethyl-, (E,E)-	94		1,921(Gómez, Ledbetter & Hartsell, 1993)
Alkene compounds	1-Propene,1,2-dichloro-	3.69	PPKPKFIWDXDAGC-UHFFFAOYSA-N	563-54-2	110.970	C_3_H_4_Cl_2_	1-Propene, 1,2-dichloro-, (Z)-trans-1,2-dichloropropene	99	–	–
	Cyclohexene, 1-methyl-4-(1-methylethylidene)-	9.78	MOYAFQVGZZPNRA-UHFFFAOYSA-N	586-62-9	136.2340	C_10_H_16_	α-Terpinolene(Z)-β-Terpinolene	98	1,093	1,097 (Kartal et al., 2007)
	1H-3a,7-Methanoazulene,2,3,4,7,8,8a-hexahydro-3,6,8,8-tetramethyl-[3R-(3α,3aβ,7β,8aα)]-	20.71	IRAQOCYXUMOFCW-KYEXWDHISA-N	469-61-4	204.3511	C_15_H_24_	Di-epi-α-cedrene2-epi-α-Funebreneα-Funebrene1,7-di-epi-α-Cedrene	96		1,403 (Walde et al., 2006)
	Benzene,1,1′-ethenylidenebis-[4-methyl-	28.12	HEDMCKGHZIRQLS-UHFFFAOYSA-N	2919-20-2	208.2982	C_16_H_16_	–	97	–	–
Phenolic compounds	Phenol, 3,5-bis(1,1-dimethylethyl)-	23.22	ZDWSNKPLZUXBPE-UHFFFAOYSA-N	1138-52-9	206.3239	C_14_H_22_O	–	98	2,310	2,310 (Shimoda et al., 1995)
	2,4-Di-tert-butylphenol	23.21	ICKWICRCANNIBI-UHFFFAOYSA-N	96-76-4	206.3239	C_14_H_22_O	–	98	1,512	1,513 (Zhao et al., 2006)
	1,4-Benzenediol, 2,5-bis(1,1-dimethylethyl)-	40.11	JZODKRWQWUWGCD-UHFFFAOYSA-N	88-58-4	222.3233	C_14_H_22_O_2_	–	98	1,457	1,457 (Khalilov et al., 1999)
	1,2-Benzenediol, 3,5-bis(1,1-dimethylethyl)-	45.39	PJZLSMMERMMQBJ-UHFFFAOYSA-N	1020-31-1	222.3233	C_14_H_22_O_2_	–	97	1,683	1,683 (Xie, Sun & Yu, 2006)
	Butylated Hydroxytoluene	23.39	NLZUEZXRPGMBCV-UHFFFAOYSA-N	128-37-0	220.3505	C_15_H_24_O	–	97	1,513	1,514 (Adams et al., 2005b)
Benzene ring compounds	1,2-Bis(trimethylsilyl)benzene	36.93	YHMJZIJXVNRXIN-UHFFFAOYSA-N	17151-09-6	222.478	C_12_H_22_Si_2_	–	91	–	–
	1,3-Bis(trimethylsilyl)benzene	5.75	RDDMOMIMRPHKSZ-UHFFFAOYSA-N	2060-89-1	222.478	C_12_H_22_Si_2_	–	91	–	–
	1,1′-Biphenyl, 2,2′,5,5′-tetramethyl-	27.88	ZHTROMYSDSTCCE-UHFFFAOYSA-N	3075-84-1	210.3141	C_16_H_18_	–	94	1,663.2	1,663.6 (Zeng et al., 2007)
	2′,3′-Dimethoxyflavone, TMS	45.71	OHEGMEUWGGBBHR-UHFFFAOYSA-N	–	282.2907	C_17_H_14_O_4_	–	87	–	–

As shown in Table 6, after the pith was subjected to a combined pretreatment process (mechanical cutting and chemical catalysis), several compounds were found in the pretreatment solution, including 9-oxabicyclo[6.1.0]nonane, cis-; 4-(trifluoromethyl)benzoic acid, 3-chloroprop-2-enyl ester; 6-hydroxy-7H-cyclohepta[b]pyridin-7-one; methyl pentadecyl ether; (Z,Z)-9,12-octadecadienal; (E)-9-octadecenal; 9-octadecen-1-ol, (Z)-; oleic acid; decyl sulphide; 9-octadecenoic acid (Z)-,2-hydroxyethyl ester; (9Z,12Z)-octadeca-9,12-dienoyl chloride; 9-octadecenoic acid (Z)-,2,3-dihydroxypropyl ester; and pentatriacont-9-ene.

**Table 6:** Chemical structure of major unknown compounds in the pretreatment solution identified from GC-MS and the linear retention index.

CAS name	Retention time (min)	IUPAC standard InChIKey	Chemical structure	Stereoisomers	CAS registry number	Molecular weight	Molecular formula	Linear retention index (LRI)	Linear retention index of literature
9-Oxabicyclo[6.1.0]nonane, cis-	10.209	MELPJGOMEMRMPL-OCAPTIKFSA-N	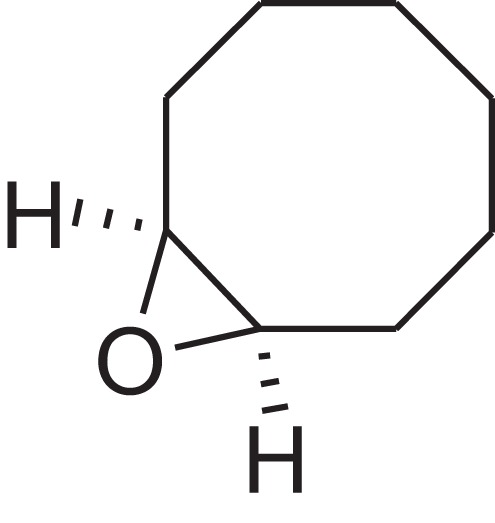	9-Oxabicyclo[6.1.0]nonane	4925-71-7	126.1962	C_8_H_14_O	983	988 (Jalali-Heravi, Zekavat & Sereshti, 2006)
4-(Trifluoromethyl)benzoic acid, 3-chloroprop-2-enyl ester	12.175	WAVIARCGALFILB-LZCJLJQNSA-N	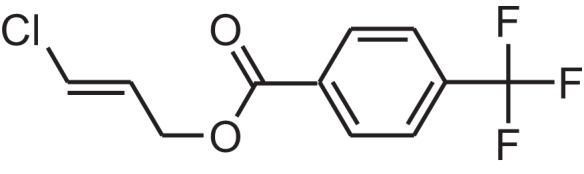	–	–	264.628	C_11_H_8_ClF_3_O_2_	1,429	1,434 (V.G. Zaikin, 2003, personal communication)
6-Hydroxy-7H-cyclohepta[b]pyridin-7-one	12.387	XAAKCCMYRKZRAK-UHFFFAOYSA-N	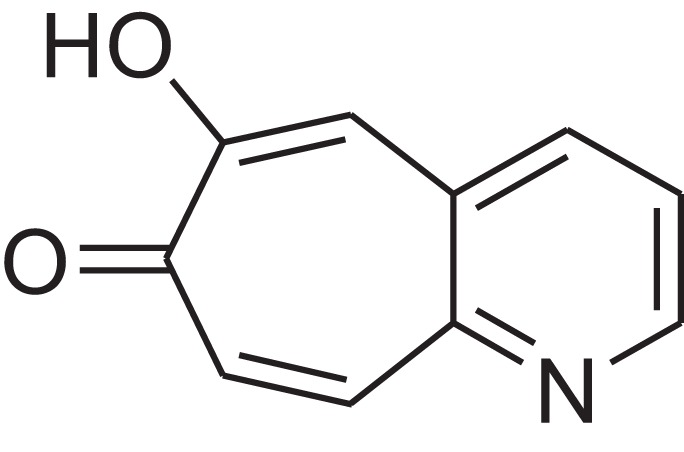	–	486-73-7	173.1681	C_10_H_7_NO_2_	1,628	1,635 (Korhonen & Lind, 1985)
Methyl pentadecyl ether	7.687	QTAFOTOCTOIEKW-UHFFFAOYSA-N	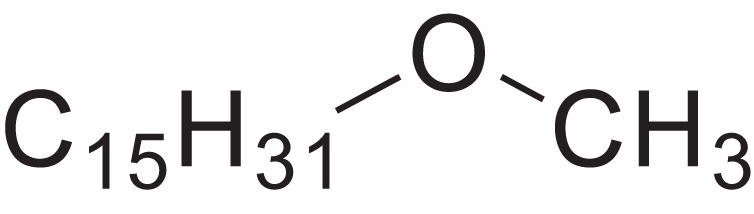	–	7307-52-0	242.4406	C_16_H_34_O	1,857	1,866 (Didaoui, Touabet & Meklati, 1997)
(Z,Z)-9,12-Octadecadienal	13.642	HXLZULGRVFOIDK-HZJYTTRNSA-N	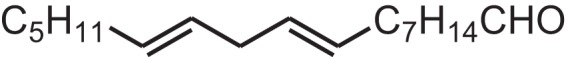	9,12-Octadecadienal	–	264.4461	C_18_H_32_O	1,977	1,971 (Sugisawa, Nakamura & Tamura, 1990)
(E)-9-Octadecenal	14.312	ZENZJGDPWWLORF-MDZDMXLPSA-N	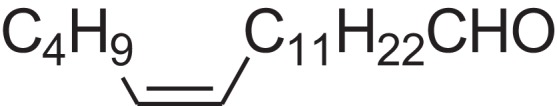	9-Octadecenal, (Z)-	–	266.4620	C_18_H_34_O	2,001	1,991 (Xie et al., 2008)
9-Octadecen-1-ol, (Z)-	14.423	ALSTYHKOOCGGFT-KTKRTIGZSA-N	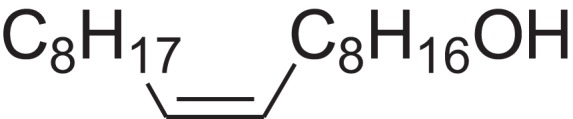	9-Octadecen-1-ol, (E)-	143-28-2	268.4778	C_18_H_36_O	2,068	2,060 (Jerkovic et al., 2010)
Oleic acid	7.687	ZQPPMHVWECSIRJ-KTKRTIGZSA-N	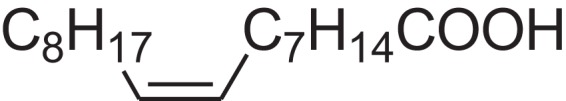	9-Octadecenoic acid, (E)-9-Octadecenoic acid	112-80-1	282.4614	C_18_H_34_O_2_	2,133	2,140 (Asuming et al., 2005)
Decyl sulphide	12.175	RKYMVQJWYYOIJB-UHFFFAOYSA-N	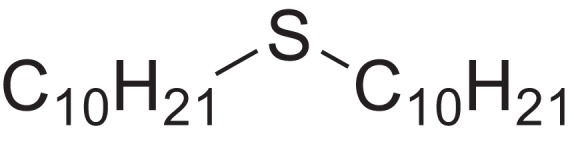	–	693-83-4	314.612	C_20_H_42_S	2,303	2,295 (Zotov, Golovkin & Golovnya, 1981)
9-Octadecenoic acid (Z)-, 2-hydroxyethyl ester	10.786	MUHFRORXWCGZGE-KTKRTIGZSA-N	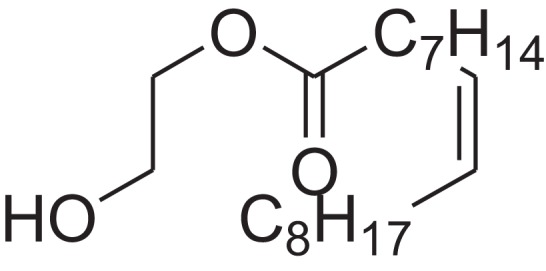	–	4500-01-0	326.5139	C_20_H_38_O_3_	2,364	2,371 (Andriamaharavo, 2014)
(9Z,12Z)-Octadeca-9,12-dienoyl chloride	11.597	FBWMYSQUTZRHAT-HZJYTTRNSA-N	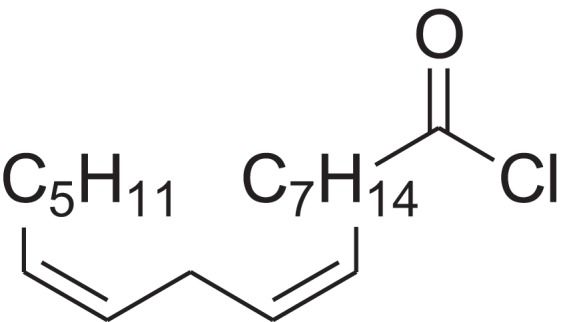	9,12-Octadecadienoyl chloride, (Z,Z)-(9E,12E)-9,12-Octadecadienoyl chloride #-	7459-33-8	298.895	C_18_H_31_ClO	2,453	–
9-Octadecenoic acid (Z)-, 2,3-dihydroxypropyl ester	11.919	RZRNAYUHWVFMIP-KTKRTIGZSA-N	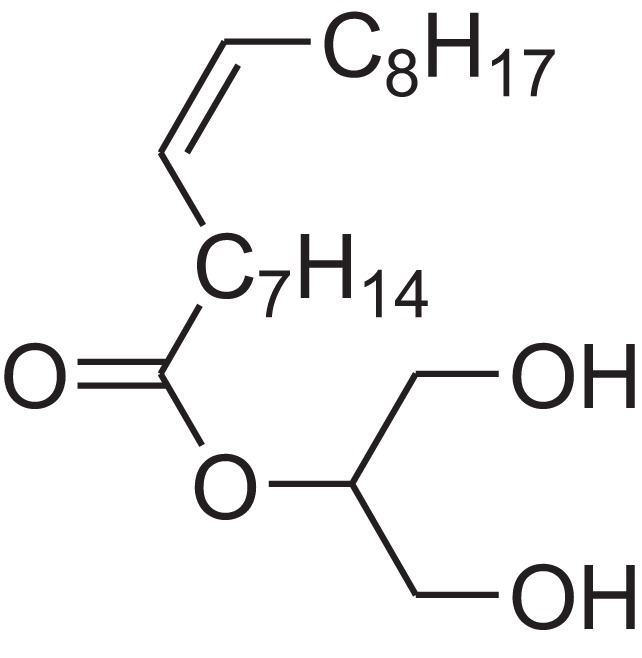	–	111-03-5	356.5399	C_21_H_40_O_4_	2,720	2,714 (Reis et al., 2000)
Pentatriacont-9-ene	39.413	AQGWMPFCRUALJK-HTXNQAPBSA-N	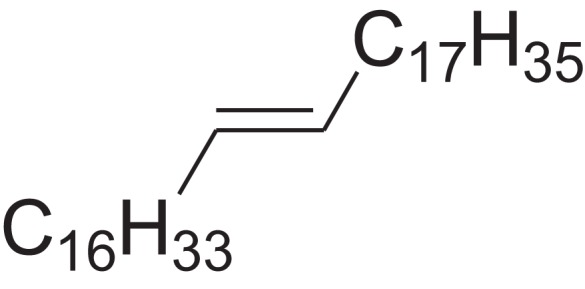	–	–	490.9303	C_35_H_70_	3,472	3,481 (Steiner, Steidle & Ruther, 2005)

## Discussion

Generally, the juice of sweet sorghum contains 1.8% fermentable sugars. SSB was found to be 45% cellulose, 27% hemicellulose and 21% lignin. However, these numeric values are affected by the harvest season. Lignocellulose consists primarily of plant cell wall materials; it is a complicated natural composite with three major constituents, that is, cellulose, hemicellulose and lignin (Wu et al., 2011). The hemicellulose–lignin complex and the crystalline structure of cellulose are largely responsible for the recalcitrance of lignocellulose to hydrolysis (Hsu, Ladisch & Tsao, 1980). Inexpensive sugars are commonly obtained from pith, the solid fibrous lignocellulosic root in SSB, and effective pretreatment is encouraged (Qin et al., 2013). Pretreatment breaks the ester and ether bonds in lignin carbohydrate complexes while increasing the internal surface area of the biomass materials (Mou, Heikkilä & Fardim, 2013). At the same time, cellulose, hemicellulose and some other compounds are released into the pretreatment solution for subsequent enzymatic hydrolysis.

Commonly used physical pretreatment methods include mechanical smashing, the application of high-energy radiation, microwave processing, pyrolysis, freezing and steam explosion. Amongst these methods, mechanical smashing is a commonly used pretreatment method for lignocellulosic materials. Before treatment, most materials are subjected to other pretreatments and must be mechanically smashed, primarily by cutting and grinding. A high-speed shearing device is used to effectively destroy the physical and chemical connections between the various components of lignocellulose. The shearing device can also destroy the crystalline structure of cellulose and increase its specific surface area. Grinding can produce cellulose with a completely amorphous structure, but this structure is highly unstable, and the crystal structure will soon reform. Smashing to greater than 400 mesh can greatly improve the enzymatic efficiency, while the associated disadvantages include a limited improvement in the overall reaction performance and large energy consumption. Moreover, excessive smashing may lead to the loss of active ingredients, including cellulose, while moderate mechanical smashing may increase the proportion of the amorphous structure of cellulose. Smashing increases the specific surface area, which benefits the efficiency of the chemical reagent and lowers energy consumption. Cellulose exposed via pretreatment is an important part of enzymatic hydrolysis and one of the essential factors that restrict the economic efficiency of enzymatic hydrolysis. The enzymes must be absorbed inside the lignocellulosic materials to degrade the cellulose. Therefore, the pith was cut to 10–20, 50 and 100 mesh in this study. It was clear that certain deposits emerged on the surface of the pith at 100 mesh (Fig. 1), which may be amorphous cellulose, released carbohydrates or other compounds. The covalent bond between hemicellulose and lignin was broken by chemical catalysis, releasing the cellulose trapped by the bond (Song et al., 2013); therefore, the structure of the pith became more porous, and new pores emerged after pretreatment (Fig. 1). These findings showed that a combined process of mechanical cutting and chemical (alkali and acid) treatment at a moderate temperature breaks the covalent bonds between hemicellulose and lignin in the complex network to remove external fibres, thereby releasing embedded cellulose for further enzymatic hydrolysis.

Alkali and acid are conventional and cost-effective pretreatments that break down the physicochemical structural and compositional factors in lignocellulosic biomass materials. They also increase the pore size to enhance enzyme penetration for improved enzymatic efficiency in biofuel production (Table 1). Zheng et al. (2018) pretreated wheat straw with 4% (w/v) NaOH at 121 °C for 1 h, which resulted in an 87.2% conversion of cellulose into glucose. Kim et al. (2012) demonstrated that after 1.2% (v/v) acid treatment of rice straw at 110 °C for 14.02 min, the subsequent enzymatic hydrolysis improved to 73.14% of the theoretical yield. The pretreatment conditions affect the composition of lignocellulosic materials and the resulting yield of sugar production. Preliminary experiments screened the appropriate parameters in this study to find the optimal particle size, temperature and chemical reagent concentrations and thus achieve the high-efficiency hydrolysis of lignocellulosic biomass. Figure 2 shows the solid recovery of pith with different pretreatments at a certain temperature. Solid recovery is negatively correlated with catalytic chemical concentration. The solid recovery also sharply decreased as the temperature increased, but excessively high temperatures led to the degradation of fermentation inhibitors and other active ingredients. A combination process at a temperature higher than 50 °C led to excess loss of cellulose and hemicellulose, which was harmful to the release of sugar and resulted in low fermentation efficiency. When the pretreatment temperature reached 100 °C, only a small amount of pretreated residues were retained after 8% (w/v) alkaline treatment. The excessive dissolution of the pith was mainly attributed to a large pore size, which increases with mesh size; under these conditions, the advantageous entry of acid and alkali solutions disintegrated the effective components under the relatively high reaction temperature (Ibbett et al., 2014; Patel et al., 2016). Additionally, the pretreated residues decreased as the pith size increased from 10–20 to 100 mesh, regardless of the pretreatment conditions, because physical smashing increased the laxity of the structure (Fig. 1), and chemical pretreatment broke the resistant structure of the pith. The solid recovery was reduced because the lignin and hemicellulose were stripped from the pith, exposing the internal structures and cellulose.

Alkali and acid treatments also had a marked effect on SSB morphology, especially the cell wall structure. Figure 1 shows images of fibre surfaces on milled samples obtained at a range of H^+^ and OH^−^-concentrations. SSB stem pith starts to lose its structure, and the fibres become detached from each other. Increasing the H^+^ and OH^−^ concentrations decreased the SSB stem pith structure further, resulting in completely unattached and independent fibres, as shown in Figs. 1D–1F. These fibres are more easily fractured than the knife-milled samples (from Figs. 1A and 1B) because their initial size is relatively large, and degradation effects are thus less significant. These samples are very useful for understanding the degradation process as a whole, but comparisons with the samples from Fig. 1D should be made cautiously, as size reduction and mechanical stress contribute to enhanced degradation. In Fig. 1C, a general view of the sample is presented, showing a conducting vessel surrounded by cell bundles that are still joined. Tissue integrity is thus maintained to some extent, but signs of degradation are evident on the surface of the wall, as shown in Fig. 1F. Delignification results in the formation of holes in the cell wall structure, and its surface therefore appears more fragile than that of the untreated samples in Figs. 1A and 1B. This difference is probably caused by the removal of lignin fractions from the inner parts of the wall as a consequence of H^+^ and OH^−^ activity. Based on these results, 50 °C, 3% (v/v) acid and 6.5% (w/v) alkali were considered the most favourable conditions for pretreatment and the most beneficial for enzymatic hydrolysis and fermentation in the next step.

Lignin is a phenolic macromolecule that is resistant to enzymatic attack and degradation, so its content and distribution are the most important factors determining cell wall hydrolysis. Lignocellulosic substrates must be pretreated to reduce this resistance and increase yield. The monomers released by enzymatic hydrolysis can be fermentable sugars. Different pretreatment methods have different mechanisms of action; they can reduce the crystallinity and/or degree of polymerization of the cellulose, increase the accessible surface area and selectively remove hemicellulose and lignin from the lignocellulosic matrix.

Cellulase is a commonly used enzyme that hydrolyses cellulose. β-Glucosidase is an enzyme with potential utility in industrial bioconversion processes because of its thermostability and high efficiency, as reported by Sørensen et al. (2011). Therefore, these two commercial enzymes, cellulase and β-glucosidase, were used to hydrolyse pith to increase the amount of released. Most of the cellulose and part of the hemicellulose in the pretreated pith were hydrolysed by the two enzymes, as indicated by the increase in total sugar yield and total sugar concentration in the hydrolysates, which increased as the mesh increased (Table 3). An appropriate enzyme ratio can improve the efficiency of hydrolysis; this result is in agreement with the findings of Marcos et al. (2013) and Cybulska, Lei & Julson (2009), who found that when steam-exploded wheat straw was hydrolysed using a high dosage of hydrolase, the cellulose and hemicellulose conversion reached 81% and 27%, respectively. This result showed that increasing the ratio between these two enzymes can, to a certain point, effectively enhance the sugar yield and ratio of hydrolysis, but an excessive enzyme ratio is not practical for the industrial treatment of straw because the cost of the process significantly increases. Therefore, a 0.67 enzyme ratio was considered appropriate for pith hydrolysis.

The D-glucose originating from cellulose can be degraded directly during hydrolysis. The C-6 hydroxyl of glucose is oxidized to D-gluconic acid, which contains a carboxyl group and was detected in this study. Hemicellulose is a highly branched heteropolysaccharide chain formed from both hexoses and pentoses, which can be decomposed into D-arabinose, L-fucose, D-galactose, D-galacturonic acid, D-mannose, D-ribose, L-rhamnose and D-xylose by pretreatment and enzymatic hydrolysis. After the acid-pretreated pith was hydrolysed, the content of the 10 neutral monosaccharides increased as the particle size increased, resulting in a maximum monosaccharide release at 100 mesh (Table 4). This finding can be attributed to increased degradation of the hemicellulose and lignin after pretreatment including both physical and chemical processes (Fig. 2). This effect resulted in increased pore size and improved the accessibility of the enzyme to cellulose, thereby increasing the release of sugar. The contents of eight neutral monosaccharides first increased with increasing pore size and later decreased, reaching their highest value in the hydrolysate at 50 mesh with alkali as the pretreatment reagent. However, gluconic acid and galacturonic acid showed a different trend from the eight monosaccharides whose content increased as the particle size increased. The reason sugar release after alkali pretreatment was optimal at 50 mesh instead of at 100 mesh was likely because lignin was heavily degraded at 100 mesh, resulting in lower solid recovery from the alkali pretreatment. The cellulose and hemicellulose were also excessively damaged, and the amorphous cellulose may have formed other substances during hydrolysis, resulting in a low content of the 10 neutral monosaccharides at 100 mesh. This finding applies to the pith derived from part of the SSB, and the sugars produced may be converted to other substances. Moreover, Sakdaronnarong et al. (2018) reported that most hemicellulose and some cellulose can be degraded into water-soluble saccharides and other small molecules during pretreatment.

How was enzymatic depolymerization stopped? In a strict mathematical sense, this process is governed by the Michaelis–Menten kinetics model. The process involves a degradation mechanism, a polymer degradation mechanism and the mathematical modelling of depolymerization kinetics. In this study, we did not analyse the mathematics behind the enzyme kinetics.

John et al. (2011) reported using NMR methods to monitor the enzymatic depolymerization of heparin. Thunberg et al. (1982) also studied the enzymatic depolymerization of heparin-related polysaccharides. Our main objective was to study the optimal time for the production of more sugar, and we refer to many reports in which 72 h is the time period used to degrade lignocellulose (Chen et al., 2018; Amiri, Karimi & Zilouei, 2014; Singh et al., 2017; Li et al., 2016). Periods longer or shorter than 72 h are not conducive to the production of sugar by hydrolysis with two enzymes. After 72 h of enzymatic saccharification, the hydrolysates were centrifuged and the liquid fraction was collected for analysis of the fermentable sugar concentration. For accuracy and reliability, all the above experiments were performed in duplicate. Cellulase is a commonly used enzyme that hydrolyses cellulose. β-Glucosidase is an enzyme with potential utility in industrial bioconversion processes because of its thermostability and high efficiency (Sørensen et al., 2011). Crystalline cellulose is attacked by several different enzymes whose concerted action releases products, and cellobiose is a major soluble product. Cellobiose is hydrolysed to glucose by β-D-glucoside glucohydrolase (β-glucosidase) (Freer, 1995). When cellulase preparations are supplemented with β-glucosidase during saccharification, glucose is the predominant product and the rate of saccharification is significantly increased (Sternbergo, Vuayakumar & Reese, 1977).

Similar to other plant cell walls, the cell wall of SSB is formed primarily from two carbohydrate moieties (cellulose and hemicellulose) embedded in a lignin matrix. Considerable research efforts have focused on the use of acid-catalysed hydrolysis to cut intrachain linkages in hemicellulose and cellulose chains contained in bagasse to produce commercial quantities of xylose, glucose and other sugars. As hydrolysis continues, the sugar may be further degraded into decomposition products such as furfural, hydroxymethylfurfural and furan resin. The acid hydrolyses hemicellulose and produces a xylose-rich liquid phase containing a small amount of lignin derivative. Therefore, acid-catalysed hydrolysis is an excellent hemicellulose recovery method (Lavarack, Griffin & Rodman, 2002; Fogel et al., 2005) and has been successfully applied to bagasse (Geddes et al., 2010; Rocha et al., 2011). Alkali treatment was originally used to increase the biomass digestibility of animal feed. Diluted alkali solution destroys lignocellulosic cell walls by dissolving hemicellulose, lignin and silica; by hydrolysing uronic acid and acetate; and by swelling cellulose (Zhang & Lynd, 2004). Lignin decomposition is usually attributed to the cleavage of α-polyphenol monomers and their polyphenolic monomers, while hemicellulose dissolution and cellulose swelling are the result of hydrogen bond weakening (Jackson, 1977). Rezende et al. (2011) determined the crystallinity of bagasse samples by X-ray diffraction before and after pretreatment. The crystallinity of their samples increased linearly with the amount of cellulose. Treating the sample with 1% H_2_SO_4_ or with 0.25% or 0.5% NaOH yielded cellulose percentages of 51%, 66% and 68%, respectively. A linear relationship was also found between these values and the crystallinity index for samples containing 100% cellulose. In our study, small deviations from this linear behaviour were observed in samples containing 80–90% cellulose (Putri, Wahyuni & Sudiyani, 2017), which corresponds to alkali treatment with 5–8% (w/v) NaOH and acid treatment with 1.5–4.5% (v/v) HCl. A slight decrease in crystallinity (2.6–4.3%) occurred in these samples relative to the results in a previous study (Vandenbrink et al., 2012; Putri, Wahyuni & Sudiyani, 2017; Hu et al., 2017). This result may indicate that severe alkali and acid pretreatments decrease the crystallinity of the sample.

Analysis of the compounds already known to be present in the pretreatment solution, especially the alcohol, ester, acid, aldehyde ketone, alkene, phenolic and benzene ring compounds, by GC/MS and the LRI detected 42 components, which accounted for 93.64% of the total peak area. Amongst these components, alcohol compounds accounted for 6.08% of the total peak area, ester compounds for 39.26%, acid compounds for 7.23%, aldehyde ketone compounds for 8.33%, alkene compounds for 2.33%, phenolic compounds for 4.45%, benzene ring compounds for 1.87% and other compounds for 24.09%. These compounds were already known, especially phenolic compounds, furans and cinnamic acid derivatives, which are the main compounds resulting from acid- and alkali-based methods. Interestingly, several major unknown compounds whose chemical structures were determined were found in the pretreatment solution, including 9-oxabicyclo[6.1.0]nonane, cis-; 4-(trifluoromethyl)benzoic acid, 3-chloroprop-2-enyl ester; 6-hydroxy-7H-cyclohepta[b]pyridin-7-one; methyl pentadecyl ether; (Z,Z)-9,12-octadecadienal; (E)-9-octadecenal; 9-octadecen-1-ol, (Z)-; oleic acid; decyl sulphide; 9-octadecenoic acid (Z)-,2-hydroxyethyl ester; (9Z,12Z)-octadeca-9,12-dienoyl chloride; 9-octadecenoic acid (Z)-,2,3-dihydroxypropyl ester; and pentatriacont-9-ene. These compounds may have been observed because the samples were analysed as received from the mill, without being washed. Therefore, they may have contained dust, soil and other debris accumulated during harvest, transportation and storage after juice extraction in the mill. These compounds may affect the decomposition of lignocellulose, as well as the release of sugar. Some of these cyclic compounds may have value and could be further extracted as a recycled resource. Moreover, this effect may accelerate the production of certain inhibitors, whose negative effects on the pith hydrolysis have not been explored. To the best of our knowledge, this study is the first to analyse these other compounds in the pretreatment pith solution using GC-MS. Our next step is to remove these compounds to increase the release of sugar and improve the utilization of lignocellulosic materials.

## Conclusion

An integrated process based on physical and chemical pretreatment effectively increased the amount of exposed cellulose to improve the saccharification rate according to SEM and HPLC detection results. In particular, the exposed cellulose and saccharification increased as the particle size increased. The crystallinity of the sample increased linearly with the amount of cellulose. The crystallinity in these samples was slightly less than that observed in a previous study, which may indicate that severe alkali and acid pretreatment decreases the crystallinity of the sample. A total of 10 types of monosaccharides were detected in this study, with a total achievable monosaccharide content of 149.48 ± 0.3 mg/g dry matter from the pith. Furthermore, compounds that were already known and unexpected compounds were analysed using GC-MS, including linear chain compounds and circular compounds. These compounds may reduce sugar production but may also be used to produce other substances for resource recycling, which makes separating these compounds the next step in our research. More importantly, this cost-effective conversion of pretreated pith will enable the efficient use of every part of sorghum bagasse for sustainable development of the economy and protection of the environment.

## Supplemental Information

10.7717/peerj.6186/supp-1 Supplemental Information 1 Data S1. Box plot of raw data from samples pretreated at 25 °C, 50 °C, 75 °C and 100 °C, respectively. Shown in Fig. 2.

10.7717/peerj.6186/supp-2 Supplemental Information 2 Data S2. Raw data of plot of model and original data under 50 °C, 50 mesh and 3 % acid, 6.5 % alkali conditions, respectively. Code for analysing the model of the data from total sugar shown in Fig. 3.

10.7717/peerj.6186/supp-3 Supplemental Information 3 Data S3. Raw data of chromatogram of standard monosaccharide solution and raw data of chromatogram of ten monosaccharides in hydrolysis solution.

## Additional Information and Declarations

### Data Availability

The following information was supplied regarding data availability: The raw data is included in Figs. 2 and 4. The raw measurements are provided in the Supplemental Files.
