# Supplementary material for: Optimization of a pretreatment and hydrolysis process for the efficient recovery of recycled sugars and unknown compounds from agricultural sweet sorghum bagasse stem pith solid waste
Source: PeerJ. 2019 Jan 10;6:e6186. doi: 10.7717/peerj.6186 (PMC6330209; doi:10.7717/peerj.6186)
Supplement: Supplemental Information 2 [file peerj-07-6186-s002.pdf]

Supplementary Information

Optimization of a pretreatment and hydrolysis process for the efficient recovery of recycled sugars and unknown compounds from agricultural sweet sorghum bagasse stem pith solid waste

Ting-Ting Jiang[1,2, †], Yan Liang[3, †], Xiang Zhou[1,*] , Zi-Wei Shi[4], Zhi-Jun Xin[1]

1. Institute of Modern Physics, Chinese Academy of Sciences, 509 Nanchang Rd., Lanzhou, Gansu 730000, People's Republic of China

2. University of Chinese Academy of Sciences, No. 19(A) Yuquan Road, Shijingshan District, Beijing 100049, People's Republic of China

3. School of Pharmacy, Lanzhou University, 199 West Donggang Road, Lanzhou, Gansu 730020, People's Republic of China

4. Gansu Agricultural University, No. 1 Yingmen Village, Anning District, Lanzhou, 730070， Gansu Province, People's Republic of China

*** Corresponding author**

Dr Xiang Zhou (syannovich@gmail.com, syannovich@impcas.ac.cn**)**

† These authors contributed equally to this work.

Fig. 3 (A) Plot of model and original data under 50℃, 50 mesh and 3 % acid conditions. Code for analysing the model of the data from total sugar.

y =Total sugar (mg/mL);
t = Ratio value of two enzymes;
t = [0.5 0.593 0.6 0.63 0.666 0.671 0.678 0.694 0.7 0.724 0.731 0.739 0.75 0.761 0.763 0.769 0.71 0.723 0.75]';
y = [0.976 0.997 1.013 1.025 1.033 1.038 1.041 1.049 1.088 1.0911 1.0962 1.0976 1.054 1.046 1.041 1.038 1.035 1.021 1.016]';

Linear model Poly2:
f(x) = p1*x^2 + p2*x + p3
Coefficients (with 95% confidence bounds):
p1 = -1.324 (-3.505, 0.8568)
p2 = 2.018 (-0.8264, 4.862)
p3 = 0.2859 (-0.6324, 1.204)
Goodness of fit:
SSE: 0.01074
R-square: 0.451
Adjusted R-square: 0.3823
Among these values, the model of the data of Fig. 3A above is given by the following equation 1: $f(x)=-1.324x^2+2.018x+0.2859$

Fig. 3 (B) Plot of model and original data under 50℃, 50 mesh and 6.5 % alkali conditions. Code for analysing the model of the data from total sugar.

Fig. 3 (A) Plot of model and original data under 50C°, 50 mesh and 3 % acid conditions. The method of weighted residuals is used for analysing the model of the data from total sugar.

y =Total sugar (mg/mL);
t = Ratio value of two enzymes;
t = [0.5 0.593 0.6 0.63 0.666 0.671 0.678 0.694 0.7 0.724 0.731 0.739 0.75 0.761 0.763 0.769 0.71 0.723 0.75]';

y = [2.473 2.538 2.563 2.571 2.886 2.872 2.856 2.861 2.816 2.823 2.829 2.831 2.836 2.821 2.817 2.798 2.763 2.699 2.832]';

Linear model Poly2:
f(x) = p1*x^2 + p2*x + p3
Coefficients (with 95% confidence bounds):
p1 = -4.993 (-11.58, 1.59)
p2 = 7.922 (-0.6625, 16.51)
p3 = -0.3055 (-3.077, 2.466)
Goodness of fit:
SSE: 0.09783
R-square: 0.6672
Adjusted R-square: 0.6256
RMSE: 0.07819
Among these values, the model of the data of Fig. 3B above is given by the following equation 2: $f(x)=- 4.993x^2+7.922x+-0.3055$
