# Supplementary material for: Optimization of a pretreatment and hydrolysis process for the efficient recovery of recycled sugars and unknown compounds from agricultural sweet sorghum bagasse stem pith solid waste
Source: PeerJ. 2019 Jan 10;6:e6186. doi: 10.7717/peerj.6186 (PMC6330209; doi:10.7717/peerj.6186)
Supplement: Supplemental Information 3 [file peerj-07-6186-s003.pdf]

Supplementary Information

Optimization of a pretreatment and hydrolysis process for the efficient recovery of recycled sugars and unknown compounds from agricultural sweet sorghum bagasse stem pith solid waste

Ting-Ting Jiang[1,2, †], Yan Liang[3, †], Xiang Zhou[1,*], Zi-Wei Shi[4], Zhi-Jun Xin[1]

1. Institute of Modern Physics, Chinese Academy of Sciences, 509 Nanchang Rd., Lanzhou, Gansu 730000, People's Republic of China

2. University of Chinese Academy of Sciences, No. 19(A) Yuquan Road, Shijingshan District, Beijing 100049, People's Republic of China

3. School of Pharmacy, Lanzhou University, 199 West Donggang Road, Lanzhou, Gansu 730020, People's Republic of China

4. Gansu Agricultural University, No. 1 Yingmen Village, Anning District, Lanzhou, 730070， Gansu Province, People's Republic of China

*** Corresponding author**

Dr Xiang Zhou (syannovich@gmail.com, syannovich@impcas.ac.cn)

† These authors contributed equally to this work.

Fig. 4 (A) Raw data of chromatogram of standard monosaccharide solution.

[min]	[Type]	[mAU*s]	[(ng/ul)/(mAU*s)]	[ng/ul]	[Name]
13.301	BB	1235.42664	1.96640e-2	24.29344	Mannose
17.095	BV	1488.10315	1.61405e-2	24.01880	Ribose
17.761	VB	899.90149	2.22013e-2	19.97902	Rhamnose
21.173	BB	1029.82788	2.04777e-2	21.08850	Gluconic acid
24.441	BB	954.92267	1.96537e-2	18.76774	Galacturonic acid
27.725	BB	1069.20532	1.88861e-2	20.19309	Glucose
31.767	BV	769.52203	3.34024e-2	25.70390	Galactose
33.119	VV	1328.41016	1.63758e-2	21.75379	Xylose
34.619	VB	1262.28113	1.62462e-2	20.50733	Arabinose
39.206	VB	918.33685	2.17410e-2	19.96560	Fucose

Fig. 4 (B) Raw data of chromatogram of ten monosaccharides in hydrolysis solution.

[min]	[Type]	[mAU*s]	[(ng/ul)/(mAU*s]	[ng/ul]	[Name]
13.377	BB	971.99304	1.96858e-2	19.13447	Mannose
16.998	BV	19.23689	2.17252e-2	4.17925e-1	Ribose
17.880	VB	146.24203	2.22559e-2	3.25475	Rhamnose
21.356	BB	463.76013	2.05921e-2	9.54978	Gluconic acid
24.627	BB	1012.54559	1.96493e-2	19.89577	Galacturonic acid
27.548	BB	4.07507e4	1.86370e-2	759.46821	Glucose
31.948	BV	1246.34314	3.31729e-2	41.34476	Galactose
33.145	VV	1.95277e4	1.66477e-2	325.09258	Xylose
34.762	VB	7620.26123	1.60670e-2	122.43485	Arabinose
39.336	VB	67.41366	1.59135e-2	1.07279	Fucose
